# Crepitus Not Necessarily a Sign of Fracture

**Published:** 1914-06

**Authors:** 


**Crepitus not Necessarily a Sign of Fracture.** De Lostalot, La Presse Med., cites an automobile accident with wound of the forearm and crepitus. X-ray showed no fracture of bone. Crepitus was due to a piece of glass impinging on bone. (Note Crepitus, though of a finer character, may also be due to inflammation in a tendon sheath).

